# Arthroscopic Repair of Meniscal Ramp Lesions in Anterior Cruciate Ligament Reconstructions: Technical Note

**DOI:** 10.1055/s-0044-1779312

**Published:** 2025-06-23

**Authors:** Felipe Galvão Abreu, Sérgio Marinho de Gusmão Canuto, Vitor Barion Castro de Pádua

**Affiliations:** 1 Department of Orthopedics and Traumatology, Hospital de Base, Faculdade de Medicina de São José do Rio Preto (Famerp), São José do Rio Preto, SP, Brazil; 2 Ortoclínica Hospital de Ortopedia, Maceió, AL, Brazil; 3 Department of Orthopedics, Faculdade de Medicina, Universidade de Marília, Marília, SP, Brazil

**Keywords:** arthroscopy, knee, meniscus, suture, artroscopia, joelho, menisco, sutura

## Abstract

Meniscal injuries are frequently associated with anterior cruciate ligament (ACL) tears. Meniscal ramp injuries involve more peripheral structures of the posterior horn of the medial meniscus (MM). Their diagnosis and repair are more challenging compared with common meniscal injuries. Although several ramp suture techniques have been described, their results remain unsatisfactory, and there are potential complications. The present technical note presents the arthroscopic repair of meniscal ramp injuries in ACL reconstruction using a posteromedial portal. Since it provides a direct view of the lesion at surgery, this is a safe technique with good outcomes.

## Resumo

Lesões meniscais são frequentemente associadas às rupturas do ligamento cruzado anterior (LCA). As lesões da rampa meniscal envolvem estruturas mais periféricas do corno posterior do menisco medial (MM) e são mais desafiadoras em relação ao seu diagnóstico e reparo em comparação com as lesões comuns do menisco. Diversas técnicas foram descritas para sutura da rampa, porém, ainda apresentando resultados insatisfatórios e com possíveis complicações. A presente nota técnica apresenta de forma clara o reparo artroscópico das lesões da rampa meniscal nas reconstruções do LCA, com auxílio de um portal posteromedial. Por proporcionar uma visão direta da lesão no momento do reparo, trata-se de uma técnica segura e com bons resultados finais.

## Introduction

Meniscal injuries are associated with anterior cruciate ligament (ACL) tears in 47% to 61% of cases, and most affect the posterior horn of the medial meniscus (MM). 1 2 3 Meniscal ramp injuries consist of longitudinal peripheral fissures of the posterior horn of the MM involving its meniscocapsular connections, the meniscotibial ligament, or both. 4 Although not recently described, ramp lesions remain a diagnostic and therapeutic challenge for orthopedists and radiologists. 5 This is partly due to the difficulty in lesion identification and repair during arthroscopy. 6 The visual field for the MM exclusively through the anterior knee compartment generates a “blind spot”, preventing the evaluation of up to 47% of the meniscal surface and hiding more posterior and peripheral injuries. This limited visual field drops to 8% of the meniscal surface when arthroscope insertion occurs through the posteromedial compartment. 7

Among the arthroscopic techniques for repairing meniscal injuries, the all-inside repair through a conventional anterior portal with implantation of meniscal suture anchor devices gained popularity due to its easy application. 8 However, in meniscal ramp lesions, fixation techniques from the inside to the outside, from the outside to the inside, or with devices using anchors added to the lack of direct injury visualization during suturing, leading to unsatisfactory results, fixation loss, and other anchor-related complications. 9 10

The present study aims to describe a suture technique for meniscal ramp injuries under direct visualization through an auxiliary posteromedial portal, as previously reported by Thaunat et al. 3 The procedure is performed during ACL reconstructions (ACLR) in patients with meniscal ramp lesions diagnosed pre- or intraoperatively.

## Surgical Technique

**Fig. 1:**
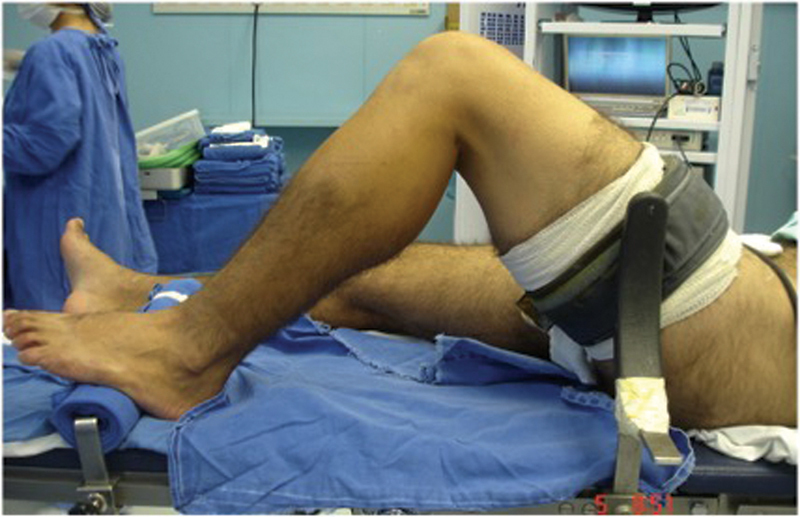
Positioning of the lower limb during the anterior cruciate ligament (ACL) reconstruction, with the foot resting on the surgical table, lateral support at the level of the tourniquet, and the knee flexed at 90°.

The patient must be in a supine position for arthroscopy, with lateral support at the level of the tourniquet and another support under the foot allowing full range of motion. The knee must be in 90° flexion on the operating table when necessary ( Fig. 1 ). Meniscal, chondral, or both injuries are addressed before ligament reconstruction. The decision on the specific graft for ACLR is based on factors from each patient and the surgeon's choice, not interfering with the approach to the meniscus.

It is possible to perform the entire surgery using a conventional 30° arthroscope. The procedure starts with the arthroscopic exploration of the knee compartments. Suspect of a ramp injury if there is any sign of meniscal instability (such as increased meniscal anterior displacement under traction) or a fissure in its lower leaflet. We routinely systematically explore the posteromedial compartment in all ACLR surgeries, as described by Sonnery-Cottet et al. 11 This exploration has three steps: (1) test the MM stability through the anterior portals; (2) visual inspection of the posteromedial compartment; and (3) the creation of a posteromedial portal to investigate a potential injury with a needle or probe.

**Fig. 2:**
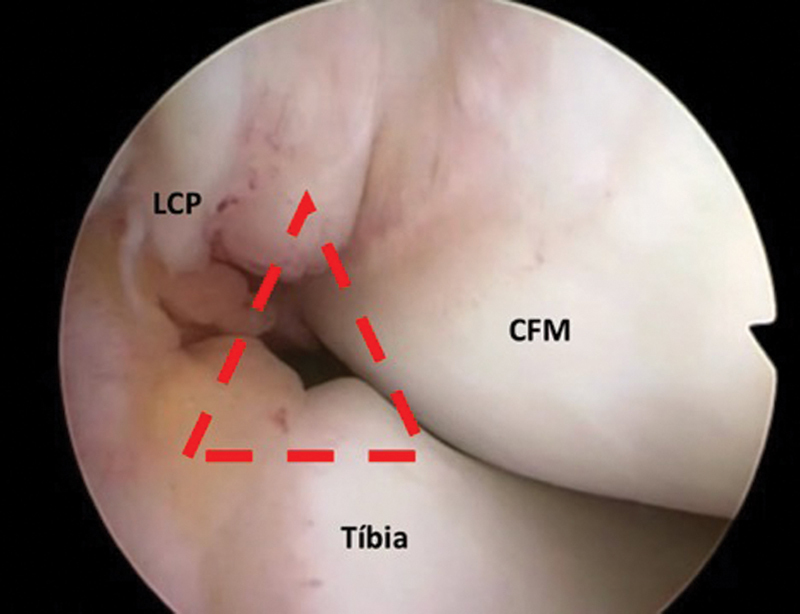
Arthroscopic image revealing the intercondylar space during arthroscope insertion to access the posteromedial compartment of the knee. The correct point is in the center of a triangle (in red) formed by the medial femoral condyle (MFC), the posterior cruciate ligament (PCL), and the tibia.

**Fig. 3:**
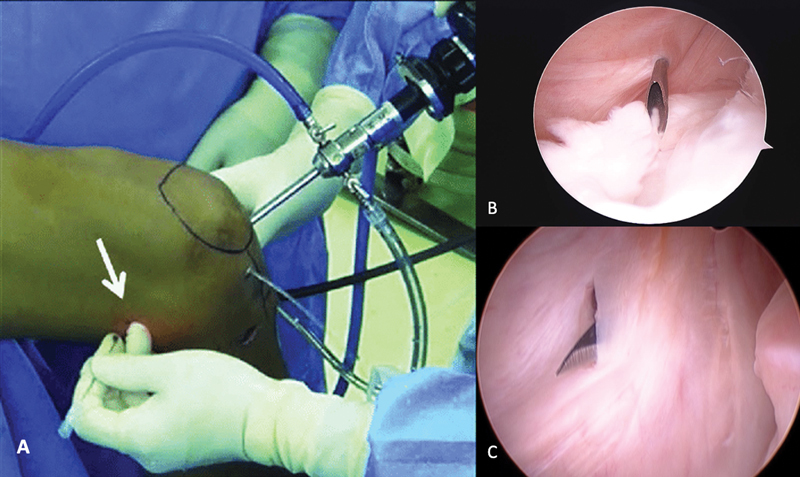
Details of the posteromedial portal confection. Transillumination prevents iatrogenic damage to vessels and nerves ( **A** ). The needle is introduced towards the lesion to define the best point for portal creation ( **B** ). The portal is made with a scalpel blade under direct visualization ( **C** ).

**Fig. 4:**
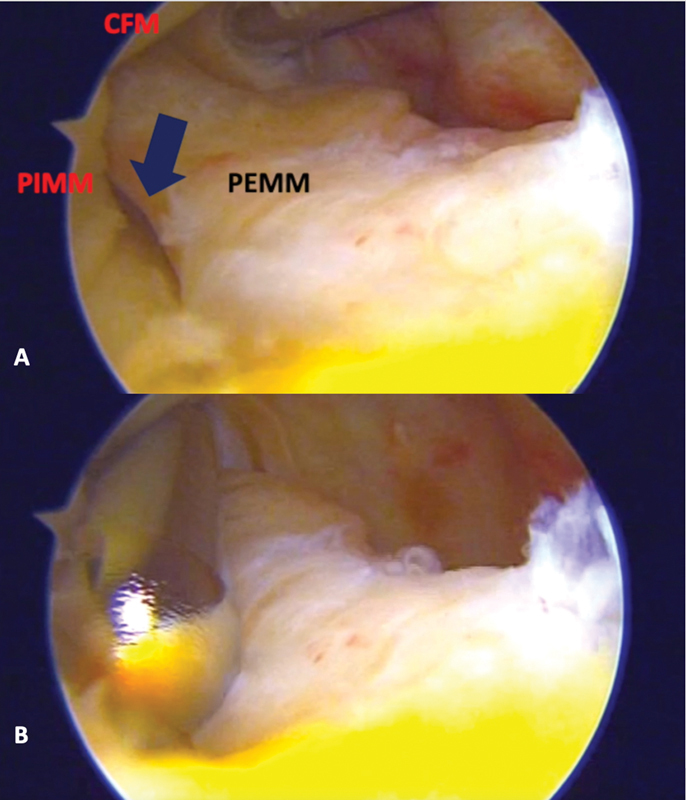
Arthroscopic image ( **A** ) showing a clear view of the meniscal ramp lesion (black arrow), the external portion of the medial meniscus (EPMM), the internal portion of the medial meniscus (IPMM), and the MFC. A shaver blade opens and smooths the meniscal ramp lesion edges ( **B** ).

The posteromedial knee compartment evaluation occurs with the arthroscope in the anterolateral portal, inserted through an intercondylar space defined by the medial femoral condyle, the posterior cruciate ligament (PCL), and the tibia ( Fig. 2 ). A valgus maneuver can facilitate arthroscope passage. Posteromedial portal creation occurs with the knee flexed at 90°. Transillumination helps to visualize veins and nerves requiring preservation. Needle introduction occurs towards the lesion, just above the flexor tendons and 1 cm posterior to the medial femorotibial joint line. Then, using a #11 blade scalpel, the incision is made under direct arthroscopic view ( Fig. 3 ). At this point, the foot is internally rotated for medial tibial condyle posteriorization, facilitating injury exposure. Next, the shaver blade is inserted through the posteromedial portal to address both lesion surfaces ( Fig. 4 ).

**Fig. 5:**
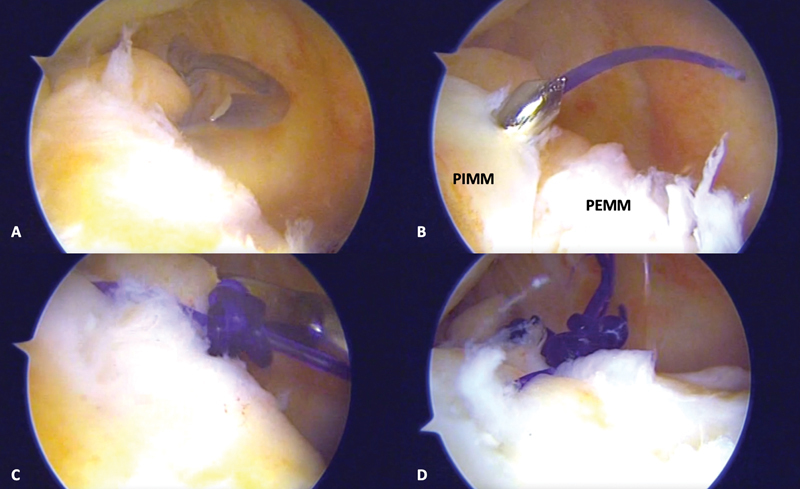
Introduction of a 25° suture hook (SutureLasso, Arthrex) through the posteromedial portal for wound repair ( **A** ). The suture hook releases the suture after crossing the EPMM and the IPMM ( **B** ). The simple suture is performed using a knot pusher ( **C** ). The final appearance of the ramp injury after repair ( **D** ).

Suturing uses a 25° suture hook (SutureLasso, Arthrex, Naples, FL, United States) at the left side for right knees and at the right side for left knees, loaded with a #1 absorbable monofilament thread (PDS; Ethicon, Inc., Raritan, NJ, United States). The surgeon manipulates the suture hook so that the sharp tip penetrates the most peripheral fragment of the lesion, containing the capsular portion, in all its thickness. Next, pass the suture hook through the central part of the MM, as described. At this point, the technique is similar to an arthroscopic repair of a shoulder with a Bankart lesion. 12 The hook releases the suture, and its free end is grasped with arthroscopic forceps and collected through the posteromedial portal. The sutures consist of any sliding knot (according to the surgeon's preference) using a knot pusher ( Fig. 5 ). Usually, 1 to 3 sutures at 1 cm intervals are enough for complete lesion repair. Confirm the satisfactory and stable repair using the probe inserted and visualized through the anterior and posteromedial portals. Finally, proceed to the ACLR procedure according to the technique chosen.

## Rehabilitation

Full knee extension and quadriceps activation are critical in early physical therapy. Instruct patients undergoing a ramp injury repair to walk with partial weight bearing with crutches for 6 weeks. Range of motion is also limited to 90° in the first 6 weeks, with a progressive increase thereafter. Exercise bike is allowed after 2 months. Allow sports that do not require directional changes (such as running and cycling) within 4 months and those requiring such changes (such as dancing and volleyball), within 6 months. Contact sports (such as football, handball, and wrestling) are allowed in 8 to 9 months. 3

## Final Comments

The failure rate in repairing posterior horn tears of the MM, including ramp injuries, remains high despite the development of all-inside suturing devices. 8 The technique described here seeks to eliminate some potential causes for these failures, bringing some advantages regarding device use. Exploration of the medial meniscus under direct visualization in the posteromedial compartment considerably improves ramp lesion diagnosis, and it provides an excellent view during repair, allowing for better edge debridement and complete injury closure control. In our practice, we often find the outermost edge of the lesion lying behind the tibial plateau during posteromedial compartment exploration. In these cases, we cannot perform any repair through the anterior knee compartment because the device will cross the internal portion of the lesion and fixate in the joint capsule, a friable, little resistance tissue, leaving the injury unrepaired. Without a direct view of the injury, we understand that repair attempts are blind. A better visualization also allows the placement of vertical sutures perpendicular to the deep fibers of the menisci, involving the meniscotibial ligament and generating better post-repair stability. This technique allows lesion reduction visualization during the procedure, which is not feasible with the all-inside implant. In addition, despite the lower cost of the 25° suture hook, the same device can do more than one suture, unlike those with anchors.

Like all surgical procedures, this one is not without disadvantages or risks. As it requires a more refined surgical technique, we understand that there is a significant learning curve. This reinforces the importance of systematizing a routine for exploring the posteromedial compartment during ACLR to familiarize the surgeon with arthroscope insertion in the posterior knee compartment and improve their sensitivity in identifying these injuries, often underdiagnosed in imaging. The additional incision and posteromedial portal creation are disadvantages of the technique. Despite the risk of saphenous nerve and vein damage, transillumination during portal construction prevents injury to these structures.
